# 4mCPred-CNN—Prediction of DNA N4-Methylcytosine in the Mouse Genome Using a Convolutional Neural Network

**DOI:** 10.3390/genes12020296

**Published:** 2021-02-20

**Authors:** Zeeshan Abbas, Hilal Tayara, Kil To Chong

**Affiliations:** 1 Department of Electronics and Information Engineering, Jeonbuk National University, Jeonju 54896, Korea; zabbas@jbnu.ac.kr; 2 Institute of Avionics and Aeronautics (IAA), Air University, Islamabad 44000, Pakistan; 3 School of International Engineering and Science, Jeonbuk National University, Jeonju 54896, Korea; 4 Advanced Electronics and Information Research Center, Jeonbuk National University, Jeonju 54896, Korea

**Keywords:** N4-methylcytosine, computational biology, neural networks, epigenetics

## Abstract

Among DNA modifications, N4-methylcytosine (4mC) is one of the most significant ones, and it is linked to the development of cell proliferation and gene expression. To know different its biological functions, the accurate detection of 4mC sites is required. Although we have several techniques for the prediction of 4mC sites in different genomes based on both machine learning (ML) and convolutional neural networks (CNNs), there is no CNN-based tool for the identification of 4mC sites in the mouse genome. In this article, a CNN-based model named 4mCPred-CNN was developed to classify 4mC locations in the mouse genome. Until now, we had only two ML-based models for this purpose; they utilized several feature encoding schemes, and thus still had a lot of space available to improve the prediction accuracy. Utilizing only a single feature encoding scheme—one-hot encoding—we outperformed both of the previous ML-based techniques. In a ten-fold validation test, the proposed model, 4mCPred-CNN, achieved an accuracy of 85.71% and Matthews correlation coefficient (MCC) of 0.717. On an independent dataset, the achieved accuracy was 87.50% with an MCC value of 0.750. The attained results exhibit that the proposed model can be of great use for researchers in the fields of biology and bioinformatics.

## 1. Introduction

In prokaryotes and eukaryotes, alterations of DNA, such as 4-methylcytosine (4mC), 5-Methylcytosine (5mC), and N6-methyladenine (6mA), play key roles in the regulation of gene expression [1,2,3]. N6-methyladenine (6mA) has recently been identified as one of the common modifications in prokaryotes; it has epigenetic functions in the regulation of chromatin organization and retrotransposons [4,5,6,7,8,9]. The modification of 5-Methylcytosine (5mC) is another popular and quite well-explored type of DNA alteration that plays a significant role in biological advancements associated with diseases like diabetes and cancer, along with several neurological disorders [10,11,12]. 4-methylcytosine (4mC) is also recognized as an effective epigenetic alteration that safeguards the self-DNA from enzyme-mediated degradation. Although 4mC has been investigated less than 5mC, it has various tasks, including DNA replication control, DNA replication error correction, cell cycle functions, and self- and non-self DNA differentiation [13,14].

To recognize the epigenetic 4mC sites, until now, several methodologies have been used, such as methylation-specific polymerase chain reaction (PCR) [15], mass spectrometry [16], whole-genome bisulfite sequencing [17], and single-molecule real-time (SMRT) sequencing [18]. These experimental methods are very costly as well as labor-intensive, and methods like SMRT frequently overestimate 4mC in prokaryotic and eukaryotic DNA [19]; therefore, cost-effective and systematic computational tools are necessary for the identification of 4mC sites in different genomes.

SMRTseq [20] was used to detect 4mC in *Mus musculus* (0.00008%), *Drosophila melanogaster* (0.904%), *Saccharomyces cerevisiae* (0.046%), and *Arabidopsis thaliana* (1.366%). However, 4mC was not detected using ultra-high-performance liquid chromatography coupled with mass spectrometry (UHPLC-ms/ms) [19] in any of these species, as the limit of the detection was set lower than 0.00005%. Using a recently created database called MethSMRT [20], certain computational tools were suggested for the prediction of 4mC sites in different species, such as *Caenorhabditis elegans, Escherichia coli, Arabidopsis thaliana, Drosophila melanogaster, Geobacter pickeringii, Rosaceae genome,* and *Geoalkalibacter subterraneus* [21,22,23,24].

To the best of our knowledge, there are only two tools available for the prediction of 4mC sites in the mouse genome—namely, 4mCpred-EL [21] and i4mC-Mouse [25]. Generally, the mouse is a well-recognized experimental animal because it has nearly the same collection of genes as humans, and it is used to replicate the effects of epigenetic changes involved in the development of mammalian diseases including humans [26,27]. Both of the above-mentioned computational tools are machine learning (ML)-based; they employ different feature encoding techniques, such as electron–ion interaction pseudopotentials (EIIP), binary profile (BPF), dinucleotide binary encoding (DPE), ring-function hydrogen chemical properties (RFHC), Kmer, and trinucleotide physio-chemical properties (TPCP), but they still have space available for improvement of the prediction accuracy, and until now, no studies have used neural networks (NNs) for the prediction of 4mC sites in the mouse genome.

In conventional machine learning, features need to be extracted by a data scientist in order to minimize the sophistication of the data and make the patterns easily apparent for the learning algorithms. On the other hand, neural networks work in a systematic way to extract the high-level features themselves from the data [28,29,30]. This removes the need for domain knowledge and the extraction of hard-core features. Therefore, we have proposed a CNN-based model for the first time for this specific mouse dataset to achieve higher accuracy for the prediction of 4mC sites. Unlike the conventional ML-based techniques, it only uses one-hot encoding and nucleotide chemical properties (NCPs) for feature extraction, and it learns high-level abstract features using the neural network architecture.

## 2. Datasets

A high-quality dataset is required to establish a sequence-based predictor for the identification of 4mC sites. We used the same dataset that was used for 4mCpred-EL [21] and i4mC-Mouse [25]. The positive samples were extracted using the MethSMRT database [20], and they contained cytosine (C) in the center. The length was fixed to 41 bp (base pairs). For the creation of an accurate model and to have a fair comparison with the previous model, 4mCPred-EL [21], we also applied the same 70% CD-HIT and omitted the sequences that displayed more than 70% similarity. Following this screening method, the positive sequences (that had 4mC sites) of the benchmark dataset were eventually collected. The same number of negative sequences (that did not have 4mC sites) were extracted randomly from the sequences that were not detected as 4mCs, and hence, a balanced dataset was acquired. After acquiring the balanced dataset, we divided it into a ratio that could be used to find the training and independent sets. Therefore, the final training set contained 746 positives (4mCs) and 746 negatives (non-4mCs), and the independent set contained 160 positives (4mCs) and 160 negatives (non-4mCs).

## 3. Proposed Methodology

Based on the benchmark dataset for mice, we developed a CNN-based model called 4mCPred-CNN. The DNA sequences in the benchmark dataset were represented in string form, such as with “AGACT…CTAAT”, with each having a length of 41 bp. Since neural networks only recognize numerical data, the strings should be transformed into numerical format before introducing them as input to the model. Both of the earlier approaches, 4mCpred-EL and i4mC-Mouse, used handcrafted features, like Kmer, MBE, EIIP, KSNC, DBE, and DPC to represent the string-like sequences in a numerical format. The handcrafted feature extraction needs a significant amount of background knowledge; therefore, rather than using all of these, we only used the one-hot encoding and NCP methods. Both of the encoding methods are explained briefly in the following subsections.

### 3.1. One-Hot Encoding

One-hot encoding is a straightforward and reliable encoding scheme that is also known as binary encoding. Using the binary representation of nucleotides, this encoder establishes sequence characteristics [31]. Nucleotides are translated into the following formats by the one-hot encoding algorithm:

$$\begin{array}{c} A:1,0,0,0\\ T:0,1,0,0\\ C:0,0,1,0\\ G:0,0,0,1 \end{array}.$$

It is then possible to transform any DNA sequence of *m* nucleotides into a vector of 4 × *m* features [32,33]. The nucleotide representation is not specific, and the A, T, C, and G representations are exchangeable.

### 3.2. Nucleotide Chemical Properties

Four groups of nucleotides make up DNA: namely, adenine (A), guanine (G), cytosine (C), and guanine (G). There are various properties of DNA, such as functional groups, ring structures, and hydrogen bonds [34,35,36]. A and G each hold two rings, while there is only one in C and T. A and T form weak hydrogen bonds with regard to secondary structures, while strong hydrogen bonds are formed by C and G. A and C make up the amino group with respect to functional groups, while G and T make up the keto group. The method for feature extraction can be described as follows:

$$a=\left\{\begin{array}{cc} 1, & n\in \{A\;,\;G\}\\ 0, & \mathrm{otherwise} \end{array}\right.$$

$$b=\left\{\begin{array}{cc} 1, & n\in \{A\;,\;T\}\\ 0, & \mathrm{otherwise} \end{array}\right.$$

$$c=\left\{\begin{array}{cc} 1, & n\in \{A\;,\;C\}\\ 0, & \mathrm{otherwise}. \end{array}\right.$$

Here, *n* denotes a nucleotide that can be translated into the following format:

A:1,1,1

T:0,1,0

C:0,0,1

G:1,0,0

For example, using this technique, a DNA sequence, “ATTCGGT”, can be translated into a vector as (1,1,1,0,1,0,0,1,0,0,0,1,1,0,0,1,0,0,0,1,0). The NCPs have similar characteristics to those of one-hot encoding, all of which can be assumed to yield distinct nucleotide representations.

**Figure 1:**
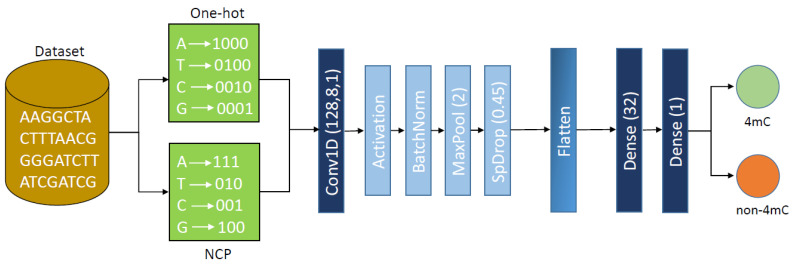
The architecture of 4mCPred-CNN.

Each sequence is translated into a matrix with 41 rows and four columns using one-hot encoding, where each column constitutes a particular DNA base of the sequence. Similarly, NCP encodes the sequences into a matrix with 41 rows and three columns. In short, only the basic composition derived using one-hot encoding and NCP comprises the data supplied to our model. Figure 1 shows the proposed model’s block diagram.

Our method uses a standard convolutional neural network consisting of a single 1D convolutional layer (Conv1D), followed by batch-normalization, pooling, and dropout layers. The Conv1D layer consists of 128 filters with a kernel size of 8. It is then followed by a linear activation function. The output of the activation function is then normalized using batch normalization (BatchNorm) to decrease the association of the results that each filter produces, which is then followed by max pooling (MaxPool) with a pool size of 2. Before using the flattening layer, we use the SpatialDropout1D (SpDrop) layer to minimize the number of parameters, with a dropout value of 0.45. The SpatialDropout1D plays the same role as the normal dropout, but rather than dropping individual nodes, it drops full 1D feature maps. If adjacent frames are closely correlated within function maps, the activations will not be regularized by normal dropouts, and will otherwise only result in an overall reduction in the learning rate. Since SpatialDropout1D can help to promote independence among function maps, we preferred to use it. After the spatial dropout, we flattened the output and fed it as input into the first dense layer, which contained 32 hidden units. We kept the same linear activation function as in the first dense layer. This dense layer was then followed by the final dense output layer, which had a single node that used a sigmoid as the activation function, which would help to classify the sequence as 4mC or non-4mC. The sigmoid activation function generated a probability score between 0 and 1. If the generated score was above 0.5, the sequence would be classified as positive or 4mC, and if the score was below 0.5, then the sequence would be classified as non-4mC or negative. Mathematically, this can be represented as:

$$Sigmoid\left(x\right)=\frac{1}{1+e^{-x}}.$$

The selected hyper-parameters were noted by using the well-known grid search algorithm.

As an optimizer, stochastic gradient descent (SGD) with a learning rate of 0.003 and a momentum of 0.8 was used in our model. The whole model was based on Keras 2.3.1 https://keras.io/ (accessed on 24 January 2021).

## 4. Evaluation Metrics

To evaluate the model’s performance and to make a fair comparison with previous methodologies, we used the same five metrics, including the accuracy, sensitivity, specificity, Matthews correlation coefficient (MCC), and area under the curve (AUC). These can be defined as:

(1) $$Accuracy=Acc=\frac{TP+TN}{TP+TN+FP+FN}$$

(2) $$Sensitivity=Sn=\frac{TP}{TP+FN}$$

(3) $$Specificity=Sp=\frac{TN}{TN+FP}$$

(4) $$MCC=\frac{TP\times TN-FP\times FN}{\sqrt{\left(TP+FP)(TP+FN)(TN+FP)(TN+FN\right)}},$$

where *TP* is for true positives, *TN* is for true negatives, *FP* is for false positives, and *FN* denotes false negatives.

## 5. Results

The proposed model was evaluated using the benchmark dataset along with an independent dataset. First, we compared our own results achieved using one-hot encoding and the NCPs on the independent dataset; then, the best approach was compared with previous methodologies.

### 5.1. One-Hot vs. NCPs

We carried out experiments using the two encoding schemes, one-hot encoding and NCPs, and compared their results, as shown in Table 1. It can be clearly seen that one-hot encoding provided a better result compared to that of the NCPs. So, we will carry out the rest of our paper using the results achieved using one-hot encoding to compare with the previous methodologies.

### 5.2. Comparison of 4mCPred-CNN with Previous Models on the Benchmark Dataset

**Figure 2:**
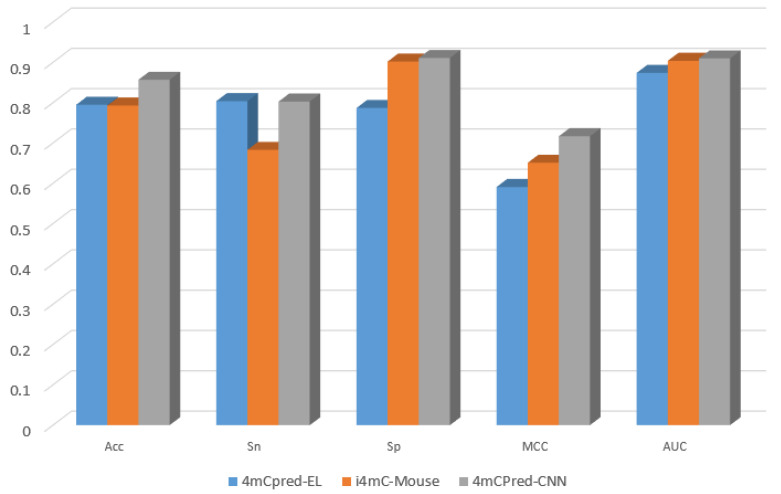
Performance comparison between 4mCPred-CNN and previous ML-based methods on the benchmark dataset—graphical representation.

To prove that our neural network based technique is superior to the previous machine-learning-based methodologies, 4mCpred-EL [21] and i4mC-Mouse [25], using the benchmark dataset, we used the same ten-fold cross-validation to get a fair comparison of the results. The results clearly depict the better performance of our model, 4mCPred-CNN, compared to the previous state-of-the-art methodologies. We used the same five evaluation metrics mentioned in Section 4 to remain consistent with the criteria of measurement seen in these studies. The outputs of 4mCpred-EL [21] and i4mC-Mouse [25] were explicitly quoted from the existing analyses. We observed that both 4mCpred-EL and i4mC-Mouse were outperformed by 4mCPred-CNN with respect to the five assessment metrics. Table 2 shows a comparison of the aforementioned existing methods with the proposed model, which outperformed 4mCpred-EL and i4mC-Mouse by 6.22 and 6.42 percentage points (p.p) for accuracy, 0.08 and 12.01 p.p for sensitivity, 12.42 and 0.92 p.p for specificity, 12.6 and 6.6 p.p for the MCC, and 3.6 and 0.6 p.p for AUC, respectively. Figure 2 provides a graphical representation of the achieved results.

### 5.3. Comparison of 4mCPred-CNN with Previous Models on the Independent Dataset

**Figure 3:**
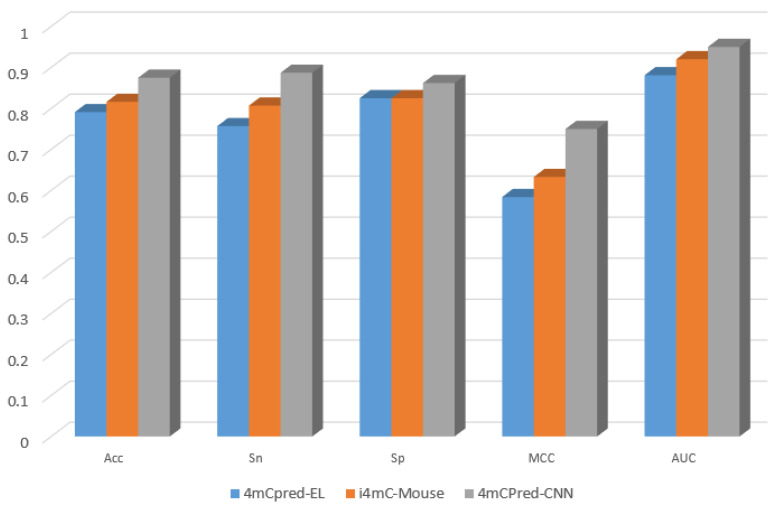
Performance comparison between 4mCPred-CNN and previous ML-based methods on an independent dataset—graphical representation.

Using the same independent dataset used for 4mCpred-EL and i4mC-Mouse, which was comprising of 160 4mCs and the same number of non-4mCs, we compared 4mCPred-CNN with these two state-of-the-art techniques and illustrated the results in Table 3. Similarly to what was found above, the outputs of 4mCpred-EL and i4mC-Mouse on the independent dataset were directly quoted from i4mC-Mouse [25], which was yielded by submitting the dataset directly to the server. The proposed model, 4mCPred-CNN, outperformed 4mCpred-EL and i4mC-Mouse by 8.4 and 5.89 p.p for accuracy, 13.03 and 8.04 p.p for sensitivity, 3.74 and 3.73 p.p for specificity, 16.6 and 11.7 p.p for the MCC, and 6.9 and 3 p.p for AUC, respectively, on the independent dataset. A graphical representation of the achieved results can be seen in Figure 3.

Our system achieved an accuracy of 87.50%, and we believe that room for improvement is still available, but the achieved result is far better than the results achieved by the techniques presented in the literature. In order to further improve the performance, we need to collect additional experimentally validated data to train a more robust model.

**Figure 4:**
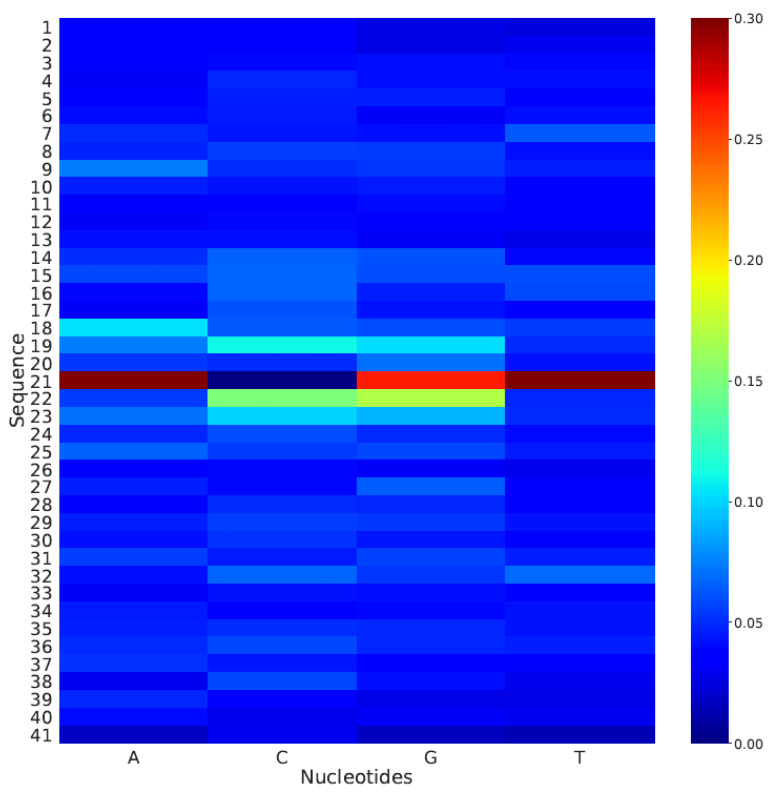
Heat map for analyzing the impacts of mutations in the model assessment.

To study the effectiveness of the model in the case of a change in DNA motifs, we applied in silico mutagenesis. We mutated the nucleotides of the available sequences so that we could predict the effect of the mutation. One after another, every nucleotide in the sequence was mutated, and the prediction was made using the proposed model. At the final stage, the average of all of the predicted scores attained for a single sequence was used to compute a heat map. Figure 4 shows the generated heat map, and it can be seen that the nucleotides at the center of the sequence had a much higher impact on the prediction than that of the nucleotides present at the edges of the sequence.

## 6. Web Server

**Figure 5:**
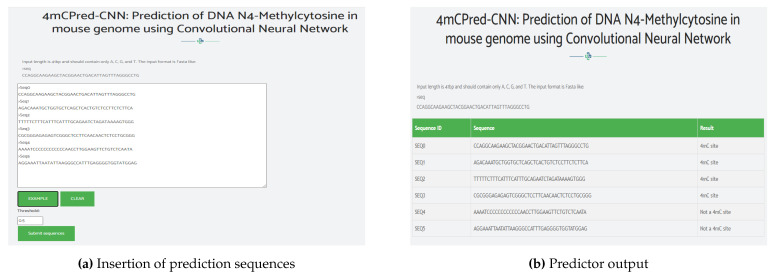
4mCPred-CNN: web server snippet.

To provide the research community with quick access to the proposed tool, a web server was created and made publicly available at http://nsclbio.jbnu.ac.kr/tools/4mCPred-CNN/ (accessed on 24 January 2021). Figure 5 shows a snippet from the web server; an example of adding prediction sequences is shown in Figure 5a, and the performance of the predictor is shown in Figure 5b.

## 7. Conclusions

In DNA modifications, 4mC plays a significant role, and it is actively engaged in controlling cell replication and levels of gene expression. Accurate detection of these sites is, therefore, an important step in identifying the particular biological processes. In order to classify 4mC sites among DNA sequences of different species, many computational models have been created by different researchers using both ML and NNs [21,23,37,38,39], but for the mouse genome, only two ML-based models are available, and no studies have used NNs for this particular species. Using NNs, we proposed a new state-of-the-art model called 4mCPred-CNN to enhance the accuracy of prediction of 4mC sites in the mouse genome. 4mCPred-CNN surpassed 4mCpred-EL and i4mC-Mouse by 8.4 and 5.89 p.p for accuracy, 13.03 and 8.04 p.p for sensitivity, 3.74 and 3.73 p.p for specificity, 16.6 and 11.7 p.p for the MCC, and 6.9 and 3 p.p for AUC, respectively, when tested on an independent dataset.

## Figures and Tables

**Table 1:** Performance comparison between one-hot encoding and nucleotide chemical properties (NCPs) on an independent dataset.

Encoding Technique	Acc (%)	Sn (%)	Sp (%)	MCC	AUC
**One-hot**	87.50	88.75	86.25	0.75	0.95
**NCPs**	83.71	81.72	85.68	0.73	0.93

**Table 2:** Performance comparison between 4mCPred-CNN and previous machine learning (ML)-based methods on the benchmark dataset.

Methods	Acc (%)	Sn (%)	Sp (%)	MCC	AUC
**4mCpred-EL**	79.50	80.40	78.70	0.591	0.874
**i4mC-Mouse**	79.30	68.31	90.20	0.651	0.904
**4mCPred-CNN**	85.72	80.32	91.12	0.717	0.910

**Table 3:** Performance comparison between 4mCPred-CNN and previous ML-based methods on an independent dataset.

Methods	Acc (%)	Sn (%)	Sp (%)	MCC	AUC
**4mCpred-EL**	79.10	75.72	82.51	0.584	0.881
**i4mC-Mouse**	81.61	80.71	82.52	0.633	0.920
**4mCPred-CNN**	87.50	88.75	86.25	0.750	0.950

## Data Availability

Data sharing not applicable. No new data were created or analyzed in this study.
